# Efficacy of acupuncture for chronic asthma: study protocol for a randomized controlled trial

**DOI:** 10.1186/s13063-015-0947-z

**Published:** 2015-09-23

**Authors:** Lei-Miao Yin, Yu Wang, Lei Fan, Yu-Dong Xu, Wen-Qian Wang, Yan-Yan Liu, Jun-Tao Feng, Cheng-Ping Hu, Pei-Yu Wang, Tie-Feng Zhang, Su-Ju Shao, Yong-Qing Yang

**Affiliations:** Shanghai Research Institute of Acupuncture and Meridian, Yue Yang Hospital, Shanghai University of Traditional Chinese Medicine, Shanghai, China; Xiang Ya Hospital, Central South University, Changsha, China; No. 3 Hospital Affiliated to Henan College of Traditional Chinese Medicine, Zhengzhou, China; Dachang Hospital, Shanghai, China

**Keywords:** Acupuncture, Chronic asthma, Efficacy, RCT, Study protocol

## Abstract

**Background:**

Although asthma symptoms can be temporarily controlled, it is recommended to use effective low-risk, non-drug strategies to constitute a significant advance in asthma management. Acupuncture has been traditionally used to treat asthma; however, the evidence for the efficacy of this treatment is still lacking. Previous clinical trials of acupuncture in treating asthma were limited by methodological defects; therefore, high-quality research is required.

**Methods/Design:**

This trial is designed as a multi-center, randomized, double-blind, parallel-group controlled trial. Patients with mild to moderate asthma will be randomly allocated to either a verum acupuncture plus as-needed salbutamol aerosol and/or prednisone tablets group or a sham acupuncture plus as-needed salbutamol aerosol and/or prednisone tablets group. Acupoints used in the verum acupuncture group are GV14 (*Da Zhui*), BL12 (*Feng Men*), BL13 (*Fei Shu*) and acupoints used in the sham acupuncture group are DU08 (*Jin Suo*), BL18 (*Gan Shu*), BL19 (*Dan Shu*). After a baseline period of 1 week, the patients in both groups will receive verum/sham acupuncture once every other day with a total of 20 treatment sessions in 6 weeks and a 3-month follow-up. The primary outcome will be measured by using the asthma control test and the secondary outcomes will be measured by using the percentage of symptom-free days, the average dosage of salbutamol aerosol and/or prednisone tablets, lung functions, daily asthma symptom scores, asthma quality of life questionnaire, and so on.

**Discussion:**

This trial will assess the effect of acupuncture on asthma and aims to provide reliable clinical evidence for the efficacy of acupuncture in treating asthma.

**Trial registration:**

ClinicalTrials.gov Identifier: NCT01931696, registered on 26 August 2013

## Background

Asthma is a noninfectious chronic disease that affects as many as 334 million people of all ages in all parts of the world [1]. The World Health Organization (WHO) has estimated that 15 million disability-adjusted life-years are lost and 250,000 asthma deaths occur around the world annually [2, 3]. Asthma is now recognized as a disease of major public health importance due to its constantly increasing prevalence, morbidity, and mortality in recent decades [2, 4].

Asthma is characterized by variable symptoms of wheeze, shortness of breath, chest tightness and/or cough, and variable expiratory airflow limitations [5]. Inhaled corticosteroids (ICS) are used as first line therapy for asthma [6]; however, a significant proportion of patients remained nonadherent to corticosteroid therapy [7]. Bronchodilators are predominant medications to treat asthma [8], but controversy exists about the risks and benefits in the application of bronchodilator drugs, such as long-acting beta-agonists [9, 10]. Regarding the potential side effects of the long-term use of conventional drugs, an effective, low-risk, and non-drug strategy would provide a valuable and adjunctive treatment in asthma management [11].

As one of the most important complementary and alternative therapies, acupuncture has been used to treat a variety of diseases for more than 2000 years [12]. The WHO listed asthma and other 42 indications for acupuncture in 1979 [13] and classified the diseases treated by acupuncture into 4 categories, 107 illnesses in 2002, including asthma [14]. The National Institutes of Health (NIH) recommended acupuncture as an adjunctive treatment in comprehensive management programs of addiction, stroke rehabilitation, asthma, etc. [12, 15]. The British Thoracic Society suggested that health care professionals should be aware of the common use of complementary and alternative medicine in asthma treatment, including acupuncture [16]. Previous studies suggested that acupuncture was effective in alleviating asthmatic symptoms and could be used as an adjunct to the conventional medical management of asthma [17]. Acupuncture also improved lung function and decreased medication dosages [18]. Our previous clinical study found that acupuncture reduced the degree and frequency of exacerbations in patients with asthma [19] and had regulatory effects on mucosal and cellular immunity in patients with allergic asthma [20].

Despite several published randomized clinical trials (RCT) evaluating acupuncture as a treatment for asthma, clear and convincing evidence has not been established [21, 22]. RCTs of acupuncture in asthma may be limited by methodological defects such as an inadequate sample size to meet statistical requirements, poor reporting with missing information, subjective bias against acupuncture and improper controls [12, 23, 24]. As a result, a high-quality RCT assessing the efficacy of acupuncture in the treatment of asthma is required. This multi-center RCT was designed to avoid the above-mentioned methodological shortcomings and aims to evaluate the efficacy of acupuncture in treating asthma.

## Methods and analysis

### Setting

This RCT will be conducted at 12 clinical centers in China: Outpatient Department of Shanghai Research Institute of Acupuncture and Meridian, Long Hua Hospital, Shu Guang Hospital, Shanghai Hospital of Integrated Traditional Chinese and Western Medicine, Shanghai First People’s Hospital, Dachang Hospital, Xiang Ya Hospital, No. 3 Hospital Affiliated to Henan College of Traditional Chinese Medicine, Zhengzhou Hospital of Traditional Chinese Medicine, Kaifeng Hospital of Traditional Chinese Medicine, Wenzhou Hospital of Traditional Chinese Medicine, Gansu Provincial Hospital of Traditional Chinese Medicine. The acupuncture doctor who treats the patients, the receptionist who is in charge of the clinical reception to select eligible patients and randomization, and assessors who collect data at these 12 clinical centers must attend a 2-day training seminar prior to the trial to ensure all practices at each of the 12 clinical centers are the same. Periodic check-ups contained the coincidence of the practices taken in every clinical center.

### Participants

#### Recruitment Strategies

There will be two strategies for patient recruitment. Participants will be recruited from the outpatient departments of the 12 clinical centers in China, and printed recruitment posters will be distributed in public clinics and nearby communities.

#### Inclusion criteria

Patients eligible for the trial must comply with all of the following:

1. Men or women, aged 14–65 years;
2. Patients with asthma history or typical clinical symptoms;
3. The diagnosis of mild and moderate asthma according to the Chinese guideline for the prevention and management of bronchial asthma [25], with increases in the forced expiratory volume in 1 second (FEV_1_) of > 12 % and > 200 mL from baseline;
4. Agree with all procedures in this trial by signing a written informed consent form.

#### Exclusion criteria

Participants meeting any of the following criteria will be excluded:

1. Participation in another clinical trial in the previous 1 month;
2. Have received systemic corticosteroids in the previous 2 weeks;
3. With systemic infection, respiratory infection, pulmonary tuberculosis and fungal infection in the previous 1 month;
4. Hospitalization due to acute exacerbation of asthma in the previous 3 months or in the baseline period;
5. Cannot stop using inhaled corticosteroids, theophylline, long-term β_2_ agonist, sodium cromoglicate, leukotriene antagonists, anticholinergic drugs or be allergic to albuterol and corticosteroids;
6. Complicated with other severe primary diseases (including hypertension, cancer, hyperthyroidism, bronchiectasis, cardiac insufficiency) and conditions that would prevent participation in the trial or put the participant at risk;
7. Women who are known to be pregnant or breastfeeding;
8. Acupuncture contraindications, such as serious atopic or infectious dermatopathy and hemorrhagic diseases (including thrombocytopenic purpura and hemophilia).

### Sample size

Sample size will be based on the primary outcome of the asthma control test (ACT). According to our pilot study, we anticipate that the improvement of the ACT will be 3.5 points in the verum acupuncture plus as-needed salbutamol aerosol and/or prednisone tablets group and 1.6 points in the sham acupuncture plus as-needed salbutamol aerosol and/or prednisone tablets group. Based on a 0.8 power to detect a significant difference (*α* = 0.05, 2-tailed), 86 patients are required for each group with a 1:1 allocation rate. To compensate for dropout patients, we plan to enroll 100 participants per group, with 200 participants in total.

### Ethics and trial registration

This trial is performed according to the principles of the Declaration of Helsinki. Ethics approval for this study was granted by the Human Research Ethics Committee of Yue Yang Hospital of Shanghai University of Traditional Chinese Medicine on 24 June 2013 (Approval number: 2013–041). The trial was registered on Clinical Trials.gov on 26 August 2013 (Registration Number: NCT01931696). Patients must provide written, informed consent before any study procedures occur.

### Procedure

An independent receptionist will initially assess each potential participant according to the inclusion and exclusion criteria. After confirmation of asthma diagnosis, participants will enter a baseline period of 1 week without treatment, during which they will be trained to use a peak flow meter, record peak expiratory flow (PEF) and maintain an asthma diary.

### Randomization

After the completion of the baseline evaluation, eligible participants will be randomly classified into two groups: the verum acupuncture plus as-needed salbutamol aerosol and/or prednisone tablets group, the sham acupuncture plus as-needed salbutamol aerosol and/or prednisone tablets group. A computer-generated, blocked random allocation sequence will be performed by Drug And Statistics software 3.2.1 [26]. The receptionist who is not involved with data collection will provide the random number and group assignment immediately by using opaque, sealed envelopes. This procedure guarantees that randomization concealment is adequate, and will not be influenced by the acupuncture doctor or participants.

### Blinding

In this trial, the receptionist is in charge of clinical reception and randomization. The acupuncture doctor only performs the treatment and will not assess the efficacy of acupuncture. According to the principle of blinding, group allocation information will be strictly withheld from all patients. Moreover, the patients, acupuncture doctors, receptionist, assessors and statisticians will be separated and any communication about the allocation information is forbidden.

### Interventions

**Fig. 1:**
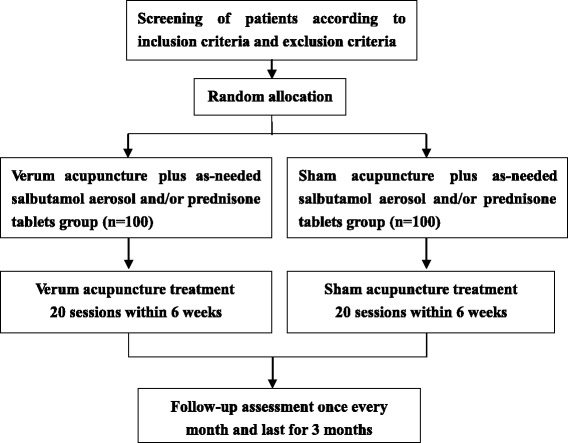
The flow chart

Acupuncture will be performed by senior attending doctors at 12 clinical centers. Asthmatic patients randomly allocated will receive verum/sham acupuncture once every other day with a total of 20 treatment sessions in 6 weeks and 3 months follow-up (see Fig. 1). Patients are required to complete case report form (CRF), asthma quality of life questionnaire (AQLQ) and record daily asthma symptom scores, acute asthma episodes and the use of as-needed salbutamol aerosol and/or prednisone tablets in the asthma diary from the completion of screening test to the end of the follow-up visits. Salbutamol aerosol (100 μg/puff) will be taken as needed for asthma attack. If the symptoms are still not controlled within a maximum safe dose, prednisone tablets will be suggested to take at a dose of 20 mg each day for 3–5 days. Patients should record use of salbutamol aerosol and/or prednisone tablets in the asthma diary.

### Verum acupuncture

The acupoints used in the verum acupuncture plus as-needed salbutamol aerosol and/or prednisone tablets group are GV14 (*Da Zhui*), BL12 (*Feng Men,* bilateral), BL13 (*Fei Shu,* bilateral). After the needle insertion, the *de qi* sensation will be induced. The twisting and lifting-thrusting manipulations are performed evenly for 20 seconds every 10 minutes and withdrawn after 30 minutes. The 0.30 mm (diameter) × 40 mm (length) disposable needle (Suzhou Medical Appliance Factory, Suzhou, China) will be used for GV14, and the 0.30 mm (diameter) × 25 mm (length) disposable needle will be used for bilateral BL12 and BL13.

### Sham acupuncture

Inappropriate acupoints for asthma treatment will be used as the sham acupuncture in the study [27, 28]. To mimic the acupuncture points in the verum acupuncture plus as-needed salbutamol aerosol and/or prednisone tablets group, we choose acupoints DU08 (*Jin Suo*), BL18 (*Gan Shu,* bilateral) and BL19 (*Dan Shu,* bilateral) in the sham acupuncture plus as-needed salbutamol aerosol and/or prednisone tablets group, which is asthma-irrelevant according to the theory of traditional Chinese medicine (TCM). To make the uniform of stimulus quantity, the same manipulation and needles will be used between the two groups.

### Outcome assessments

#### Primary outcome measurement

The efficacy of acupuncture for chronic asthma is assessed by the following primary outcome which measures the change in ACT [29].

#### Secondary outcome measurements

1. Percentage of symptom-free days (SFDs) [30];
2. Average dosages of salbutamol aerosol and/or prednisone tablets [31, 32];
3. Lung functions assessed by FEV_1_ and mean morning and evening PEF [33, 34];
4. Daily asthma symptom scores [35];
5. AQLQ [36];
6. Peripheral blood eosinophil (EOS) counts;
7. Number of asthma exacerbations.

#### Follow-up

Follow-up assessments will be conducted at a clinic visit once every month and last for 3 months. Patients are required to complete the CRF, AQLQ and assess lung functions. To encourage participant compliance, we will show special solicitude for every participant and closely monitor the evolution of their illness.

### Statistical analysis plan

#### Data integrity

All records will be collected in the original data source. Primary entries are not allowed to be changed and any correction should be explained by the responsible acupuncture doctor with a signature in the appended notes. The CRF files will be collected after the verification and data input will be done separately by two data collectors, which will be locked once the checking work is done.

#### Methods of statistical analyses

The modified intent-to-treat (mITT) analysis set will be considered as the full analysis set. This will include all evaluable patients: ie, all randomized patients who receive at least one treatment and who have a score at randomization and at least one post-randomization ACT total score. The mITT analysis set will be used for efficacy analyses. Missing data will be replaced according to the principle of LOCF (the last observation carried forward). The per-protocol (PP) analysis set will include only those mITT patients who have no significant protocol deviations and who received the treatment to which they were randomized. Analysis of primary efficacy endpoint will be repeated on the PP analysis set to test for robustness of results. All the randomized patients who receive at least one treatment and for whom any post-baseline safety data are available will be included in the safety analysis set. The safety analysis set will be used to assess safety variables.

In general, all efficacy and safety variables will be summarized using descriptive statistics and graphs as appropriate. Continuous variables will be summarized by descriptive statistics (sample size (*n*), mean, standard deviation (SD), minimum, median and maximum). Categorical variables will be summarized in frequency tables (frequencies and percentages). The primary efficacy variable, the change from baseline in ACT total score to the end of treatment period will be analyzed using an analysis of covariance (ANCOVA). The ANCOVA analysis will include the baseline ACT total score as covariate, treatment as a fixed effect and center as a random effect. Secondary endpoints will be analyzed using a *t* test or Wilcoxon rank sum test as appropriate. All analyses will be performed using SAS software, version 9.3 (SAS Inc., Cary NC, USA). All statistical tests will be conducted at a 2-tailed significance level of 5 %.

#### Safety evaluation

Adverse events are defined as unfavorable or unintended signs, symptoms or diseases occurring after the treatment that are not necessarily related to the acupuncture intervention. Any adverse event or abnormality will be recorded in the CRF regardless of relationship to the study intervention. If any serious adverse events occur, the intervention will be stopped immediately and appropriate action will be taken. This will be reported promptly to the institutional review board, according to the protocol.

## Discussion

Acupuncture has traditionally been used to treat asthma in China and has been shown to be beneficial in some aspects of asthma [37], but a previous Cochrane review concluded that there was not enough evidence to make recommendations about the value of acupuncture in asthma treatment due to poor trial quality, which needed further research [38]. However, the document of “British guideline on the management of asthma” explained and recognized that lack of evidence does not necessarily mean ineffectiveness of the treatment, which required high-quality research to support the recommendation [39]. In this randomized controlled trial, we aim to evaluate the effect of acupuncture in treating asthma and provide reliable clinical evidence without bias.

Compared to previous RCTs of acupuncture in treating asthma [38], the strengths of our trial are that it is multi-center, with strictly concealed randomization, rigorous blinding, large sample size, and proper selection of ACT and AQLQ for evaluating acupuncture efficacy. The acupoints GV14 (*Da Zhui*), BL12 (*Feng Men*), BL13 (*Fei Shu*) were selected based on the theory of TCM and were popularly used in treating asthma in China [20], which also had supports from our biological studies [37, 40]. For the sham acupuncture, irrelevant acupoints were used with the same manipulation as the verum group. Because Chinese patients are seldom acupuncture-naïve and most are considerably familiar with acupuncture procedure and feeling, this means that it is unfeasible to apply a non-invasive approach. One of the possible limitations in this trial is the unpredictable stress induced by the sham acupuncture, which may have certain effect on asthma.

In summary, the efficacy of acupuncture on asthma will be carefully assessed and the trial aims to provide reliable clinical evidence for the efficacy of acupuncture in treating asthma.

## Trial status

The trial is currently ongoing. Participant recruitment started in August 2013, and is expected to end in July 2015.

## Abbreviations

ACT: asthma control test
ANCOVA: analysis of covariance
AQLQ: Asthma Quality of Life Questionnaire
CRF: case report form
EOS: eosinophils
FEV_1_: forced expiratory volume in 1 second
ICS: inhaled corticosteroids
LOCF: last observation carried forward
mITT: modified intent-to-treat
NIH: National Institutes of Health
PEF: peak expiratory flow
PP: per-protocol
RCT: randomized clinical trials
SD: standard deviation
SFDs: symptom-free days
TCM: traditional Chinese medicine
WHO: World Health Organization
